# Functional genomic insights into *Floricoccus penangensis* ML061-4 isolated from leaf surface of Assam tea

**DOI:** 10.1038/s41598-025-86602-x

**Published:** 2025-01-23

**Authors:** Patthanasak Rungsirivanich, Elvina Parlindungan, Jennifer Mahony, Witsanu Supandee, Narumol Thongwai, Douwe van Sinderen

**Affiliations:** 1 Department of Biology, Faculty of Science, Chiang Mai University, Chiang Mai, 50200 Thailand; 2 Community Development Department, Ministry of Interior, Bangkok, 10210 Thailand; 3 School of Microbiology, University College Cork, Cork, T12 YT20 Ireland; 4 APC Microbiome Ireland, University College Cork, Cork, T12 TP07 Ireland; 5 Engineering Science Classroom, King Mongkut’s University of Technology Thonburi, Bangkok, 10150 Thailand

**Keywords:** Functional genomics, Bacterial genomics, Bacterial genetics, Comparative genomics

## Abstract

*Floricoccus penangensis* ML061-4 was originally isolated from the leaf surface of an Assam tea plant (*Camellia sinensis* var. *assamica*) from Northern Thailand. To assess the functions encoded by the *F. penangensis* ML061-4 genome, gene identification and annotation were undertaken by in silico analysis. The complete genome of *F. penangensis* ML061-4 consists of single chromosome of 2,159,127 base pairs, containing a GC content of 33.2% and encompassing 2049 predicted protein-encoding genes. A total of 1195 genes (58.0%) in the *F. penangensis* ML061-4 genome have assignable functions based on BlastKOALA analysis. Furthermore, 1235 genes (59.9%) were classified into six KEGG functional categories with 187 associated pathways, while 1419 genes (68.8%) were assigned a putative function by the Clusters of Orthologous Groups (COGs) database. The ML061-4 genome was evaluated for genes associated with complex carbohydrate metabolism, bacterial adhesion, virulence factors, pathogenicity, bacteriophages, antiviral defence systems as well as toxin- and antibiotic-resistance associated genes, and genes involved in toxin production, secondary metabolite biosynthesis and xenobiotics biodegradation. The obtained results support the notion of *F. penangensis* ML061-4 being safe for biotechnological and food industry purposes. This is the first report outlining functional genomic insights regarding a member of the genus *Floricoccus*.

**Supplementary Information:**

The online version contains supplementary material available at 10.1038/s41598-025-86602-x.

## Introduction

*Floricoccus penangensis*, a lactic acid bacterium, was first described by Chuah et al.^1^. It has been classified belonging to the family *Streptococcaceae* originally isolated from flowers of *Durio zibethinus*. The genus *Floricoccus* was explicitly separated from the genera *Lactococcus* and *Streptococcus*, with which it exhibits 94–99% and 49–76% sequence identity, as based on 16S rRNA and the housekeeping *rpoB* genes, respectively^1,2^. *Floricoccus* members have not only been found in various environmental locations, such as plants, flowers and foods^1,2^, but have also been associated with potential eukaryotic hosts, such as animals (as a component of the fecal/gut microbiome)^3^ and humans (respiratory secretions)^4^. Despite their frequent isolation, the precise ecological roles and functions of members of the genus *Floricoccus* are still largely unknown.

Various members of the genera *Lactococcus* and *Streptococcus*, which are phylogenetically closely related to *Floricoccus*, are extensively used for the production of fermented foods, such as yoghurt, cheese, kefir and lactic butter^5,6^. For example, *Lactococcus raffinolactis* is widely used as a starter culture in fish, meat, vegetables and milk fermentations^7^, while *Streptococcus thermophilus* strains are employed in the production of yogurt and certain cheeses and have also been reported to exhibit probiotic potential^8^. However, there have been reports on lactococcal and streptococcal isolates acting as pathogenic bacteria or opportunistic pathogens in humans and animals. For instance, *Streptococcus suis* can act as the causative agent of arthritis, meningitis and septicemia^9^, while *Lactococcus lactis* has been reported to very occasionally cause endocarditis and liver abscesses in humans. Moreover, *Lactococcus garvieae* is described as a fish pathogen, whereas *Lactococcus petauri* is associated with abscesses in animals so also exerts pathogenic potential^10^.

*F. penangensis* strain ML061-4 is a Gram-positive, coccus-shaped, non-endospore forming, non-motile and catalase-negative bacterium. This strain has been reported to exhibit properties such as acid-base tolerant, ability to aggregate and adhere to human epithelial cells, which may be desirable in a probiotic exploitation context^2^. In addition, *F. penangensis* ML061-4 may act as a biological control agent in Assam tea (*Camellia sinensis* var. *assamica*) cultivation and during tea leaf fermentation through its ability to produce organic acids as a result of fermentation of a variety of carbohydrates, such as glucose, fructose, mannose, *N*-acetyl-glucosamine, arbutin, aesculin, maltose, sucrose, trehalose, starch and glycogen^1,11^.

Although *Floricoccus* has been described as a novel genus, including species such as *F. penangensis* and *F. tropicus*, and being related to streptococci and lactococci, floricoccal genomes have not been extensively analyzed. The genome sequence of *F. penangensis* JCM 31735^T^ was previously reported by Chuah et al.^1^. using an Illumina sequencing platform. Illumina sequencing allows low-cost and rapid genome analysis, however, this sequencing technology typically results in the generation of multiple contigs, thus not leading to a fully assembled, high quality genome sequence since it is based on the assembly of short reads^12,13^. A recent study by Rungsirivanich et al.^14^. described a completed genome sequence of *F. penangensis* ML061-4 using a combination of Illumina and Pacific Biosciences (PacBio) sequencing platforms. PacBio sequencing generates long reads and facilitates assembly of reads into fully circular chromosomes (and plasmids where relevant)^15^.

In silico analysis has been applied for gene predictions to better understand the genetic and metabolic potential as well as to predict functional characteristics of strains^16,17^. In the present study, we analyze and characterize the genome of *F. penangensis* ML061-4 and perform an extensive gene identification and annotation effort, combined with comparative genome analysis including genomes from floricoccal, lactococcal and streptococcal groups in order to validate the functionality and corroborate safety aspects of *F. penangensis* ML061-4.

## Results and discussion

### General genome features of *F. penangensis* ML061-4

In a previous study, the complete genome of *F. penangensis* ML061-4 was reported to constitute a single contig of 2,159,127 base pairs with a 33.2% GC content, and to encompass 2,134 predicted genes comprising 19 rRNAs, 63 tRNAs, 2 ncRNAs, 16 pseudogenes and 2,049 protein-encoding genes^14^. The genome size of *F. penangensis* ML061-4 is within the range of those determined for three other *Floricoccus* species (2.05–2.16 Mbp), or to those of *Lactococcus* (1.96–2.42 Mbp) and *Streptococcus* (2.15–2.47 Mbp) species (Table 1). Genomes belonging to members of the floricoccal group exhibit a lower % G + C content (approximately 33%) when compared to those of lactococci (35–38%) and streptococci (39–42%). To validate genetic relatedness among members of these three genera, the average nucleotide identity (ANI) value of strain ML061-4 was determined and compared to type strains representing members of the genera *Floricoccus*, *Lactococcus* and *Streptococcus*. Strain ML061-4 exhibits ANI values of 97.90, 93.70, 69.98 and 70.16% when compared with *F. penangensis* JCM 31735^T^, *Floricoccus tropicus* JCM 31733^T^, *Lactococcus plantarum* DSM 20686^T^ and *Streptococcus salivarius* NCTC 8618^T^, respectively (Table 1). Congruently, the 16S rRNA gene sequence of strain ML061-4 displayed 100.0 and 99.8% identity to that of *F. penangensis* JCM 31735^T^ and *F. tropicus* JCM 31733^T^, respectively, as previously described by Rungsirivanich et al.^2^.

**Table 1:** Genome features of *F. penangensis* ML061-4 compared with *Floricoccus* spp., *Lactococcus* spp. and *Streptococcus* spp.

Feature	*F. penangensis* ML061-4	*F. penangensis* JCM 31735^T^	*F. tropicus* JCM 31733^T^	*L. plantarum* DSM 20686^T^	*L. lactis* DSM 20481^T^	*L. taiwanensis* NBRC 109049^T^	*S. salivarius* NCTC 8618^T^	*S. hillyeri* DSM 107591^T^	*S. ovuberis* CCUG 69612^T^
Size (bp)	2,159,127	2,049,722	2,157,756	1,989,191	2,421,471	1,956,764	2,188,923	2,147,614	2,467,419
ANI values^a^	Not relevant	97.90	93.70	69.98	69.84	69.34	70.16	69.95	68.84
G + C content (mol%)	33.2	33.1	33.0	36.8	35.1	38.7	40.1	39.5	42.8
No. of plasmids	0	NA	NA	NA	0	NA	0	NA	NA
Genes (total)	2134	2091	2243	1914	2356	1908	2055	2079	2353
CDSs (total)	2049	2032	2179	1877	2268	1846	1965	2020	2304
No. of rRNAs	19	5	6	3	19	7	18	4	5
No. of tRNAs	63	51	55	30	65	51	68	50	40
No. ncRNAs	2	3	3	4	4	4	4	4	4
Pseudogene	16	25	16	44	12	21	58	63	143
Contigs	1	31	26	43	1	17	1	83	48
Origin	Fresh Assam tea leaf	Flower (*Hibiscus rosa-sinensis* L.)	Flower (*Durio zibethinus* L.)	Frozen peas	Water in the drain pit of a kitchen sink	Fresh cummingcordia	Oral cavity	Tracheal (*Equus ferus caballus*)	Subcutaneous abscess in the udder of a sheep
Sequencing Technology	PacBio RSII	Illumina MiSeq	Illumina MiSeq	Illumina HiSeq	ABI 3730xl	Illumina NovaSeq	Sanger dideoxy sequencing	Illumina	Illumina NovaSeq
Assembly level	Complete	Draft	Draft	Draft	Complete	Draft	Complete	Draft	Draft
Genbank accession number	CP075561	NZ_MKIQ00000000	NZ_MKIR00000000	NZ_JXJX00000000	NC_020450	NZ_BNDT00000000	NZ_CP009913	NZ_RCVM00000000	NZ_JAAXPR000000000

All data obtained from NCBI (https://www.ncbi.nlm.nih.gov/) except for strain ML061-4, ^a^Average nucleotide identity (ANI) values obtained from EZBioCloud database (https://www.ezbiocloud.net/tools/ani). NA, not available. CDSs, coding sequences.

### Genetic fingerprint and whole-genome phylogenetic analysis

**Fig. 1:**
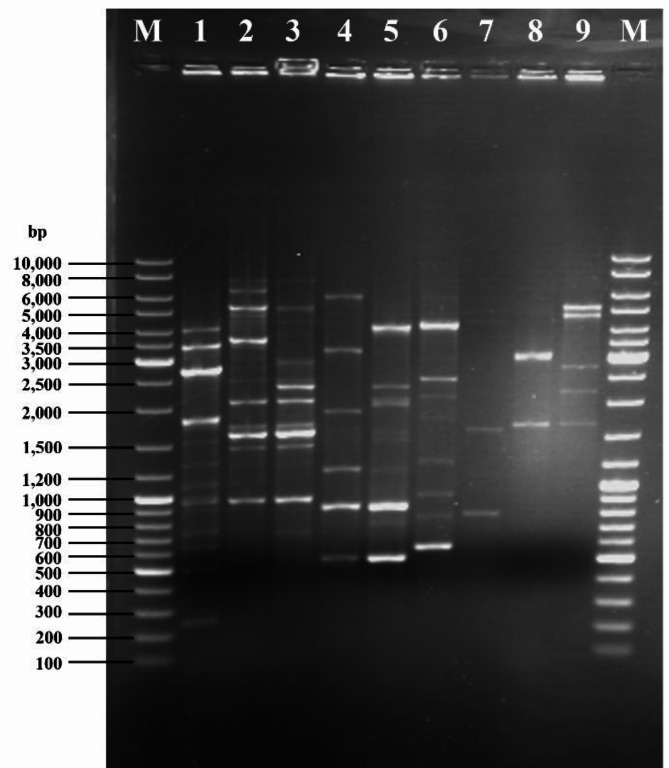
(GTG)5-PCR DNA fingerprints of *Floricoccus penangensis* ML061-4 compared with *Streptococcus* spp., *Lactococcus* spp. and *Bacillus* spp. Lane M : GeneRuler DNA Ladder Mix, Lane 1 : *F. penangensis* ML061-4, Lane 2 : *S. thermophilus* Rico66, Lane 3 : *S. thermophilus* Brie28, Lane 4 : *L. lactis* Tempeh6L, Lane 5 : *L. lactis* RMN5F, Lane 6 : *B. velezensis* ML122-2, Lane 7 : *B. licheniformis* ML075-1, Lane 8 : *B. cereus* TISTR 687, Lane 9 : *B. subtilis* NCTC 10073.

DNA fingerprint (GTG)_5_ profiles showed that *F. penangensis* ML061-4 is closely related to the genera *Streptococcus* and *Lactococcus*. The DNA fingerprint of strain ML061-4 included a band with a size of approximately 1,000 bp which it has in common with the fingerprint obtained for *S. thermophilus* Rico66 and *S. thermophilus* Brie28. The same fingerprinting experiment of *F. penangensis* ML061-4 also generated bands with sizes of ~ 3,500 and ~ 4,000 bp, which corresponds to results obtained for *L. lactis* Tempeh6L and *L. lactis* RMN5F, respectively. The obtained results also support the phylogenetic closeness between members of the genera *Streptococcus* and *Lactococcus* as illustrated by the generation of very similarly sized products of 2,100, 2,400 and 2,500 bp. In contrast, and as expected based on its distant phylogenomic relationship, the genetic fingerprinting profiles obtained *Bacillus* species were clearly distinct from each other and from those obtained for floricoccal, lactococcal and streptococcal members (Fig. 1).

**Fig. 2:**
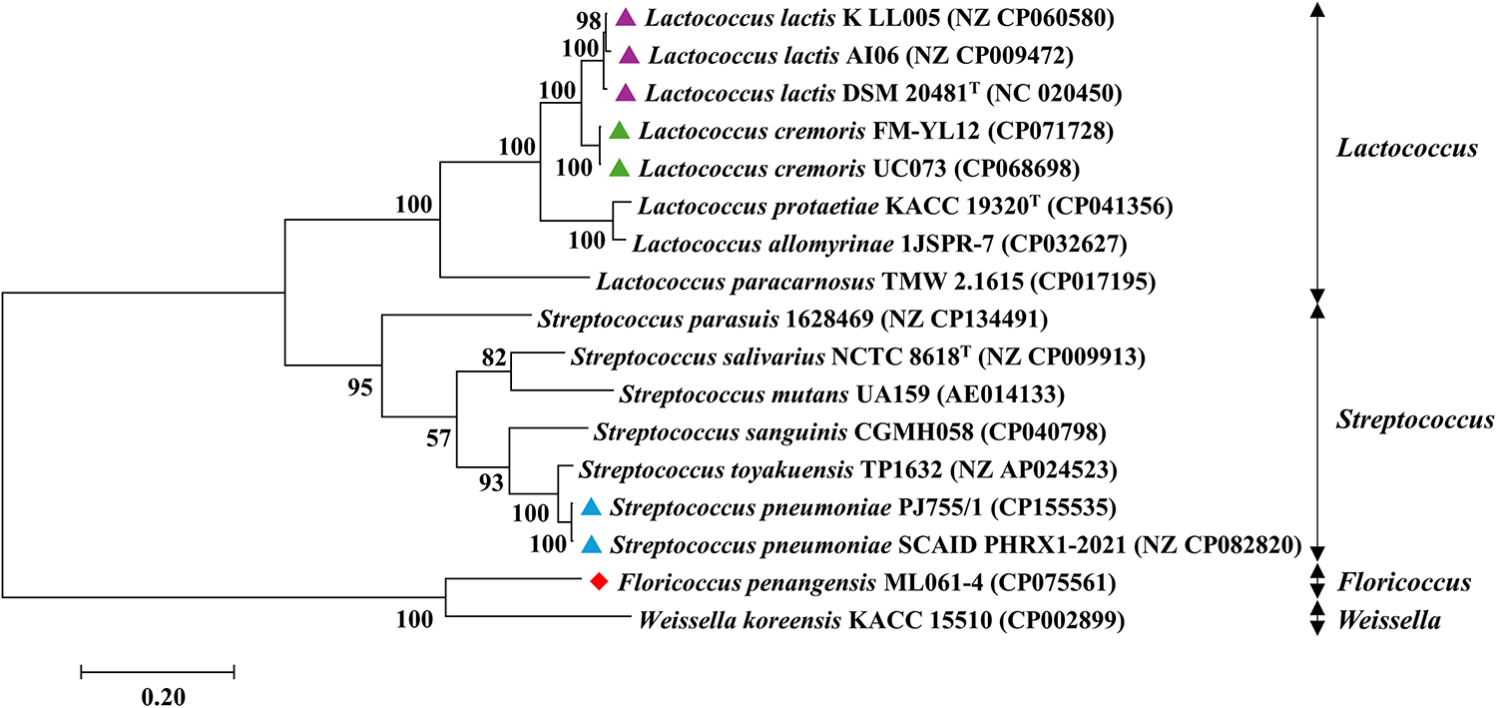
Whole genome sequencing (WGS) phylogenetic tree of *Floricoccus penangensis* ML061-4 (red diamond) and the members of the genera *Lactococcus* and *Streptococcus*. The phylogenetic tree consisting of 17 strains was constructed using the Maximum Likelihood method. Bootstrap values were generated using 1,000 replications. The WGS of *Weissella koreensis* KACC 15510 was used as outgroup. Different symbols were used to highlight different species: a purple triangle corresponds to *Lactococcus lactis*, a green triangle indicates *Lactococcus cremoris* and a blue triangle reflects *Streptococcus pneumoniae*.

A phylogenomic tree was constructed using complete genome sequences of *Lactococcus* spp. (8 genomes) and *Streptococcus* spp. (7 genomes) as well as that of *F. penangensis* ML061-4. The genome sequence of *Weissella koreensis* KACC 15510 was used as outgroup. The obtained phylogenomic tree showed that *F. penangensis* ML061-4 genome is clearly distinguished from genomes belonging to members of the genera *Lactococcus* and *Streptococcus*. Average nucleotide identity (ANI) between the genome of *F. penangensis* ML061-4 and those of selected species of *Lactococcus* and *Streptococcus* was determined. Strain ML061-4 was shown to elicit ANI values of 70.09, 69.43 and 69.90% when compared with *L. cremoris* FM-YL12, *L. protaetiae* KACC 19320 and *S. pneumoniae* PJ755/1, respectively. Furthermore, *F. penangensis* ML061-4 exhibits ANI value of 67.05% when compared with *W. koreensis* KACC 15510. The phylogenetic tree analysis of the whole genome is presented in Fig. 2.

### Genome annotation of *F. penangensis* ML061-4

**Fig. 3:**
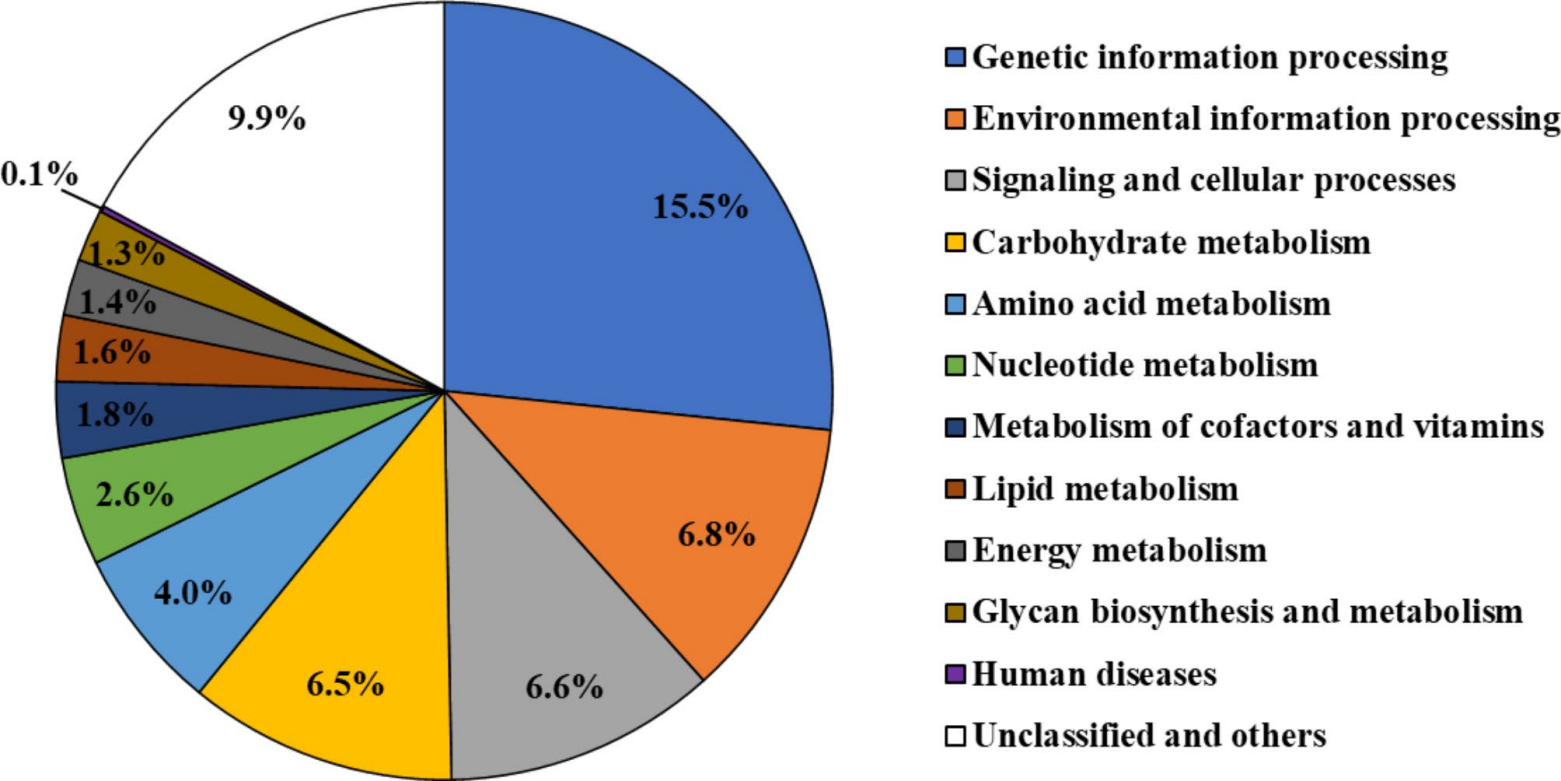
Gene functional categories of *Floricoccus penangensis* ML061-4 genome predicted by BlastKOALA.

Functional assignment of the gene content of *F. penangensis* ML061-4 was possible for 1,195 genes (58.0%) involving 187 pathways as predicted by BlastKOALA; these annotations were shown to relate to genetic information processing (15.5%, 319 genes), environmental information processing (6.8%, 141 genes), signaling and cellular processes (6.6%, 136 genes), carbohydrate metabolism (6.5%, 134 genes), amino acid metabolism (4.0%, 82 genes), nucleotide metabolism (2.6%, 54 genes), metabolism of cofactors and vitamins (1.8%, 38 genes), lipid metabolism (1.6%, 33 genes), energy metabolism (1.4%, 28 genes), glycan biosynthesis and metabolism (1.3%, 26 genes), human diseases (0.1%, 3 genes) and unclassified (9.9%, 201 genes) (Fig. 3; Supplementary Table S1). These metabolic pathways may allow strain ML061-4 to survive under nutrient poor conditions^18^.

**Fig. 4:**
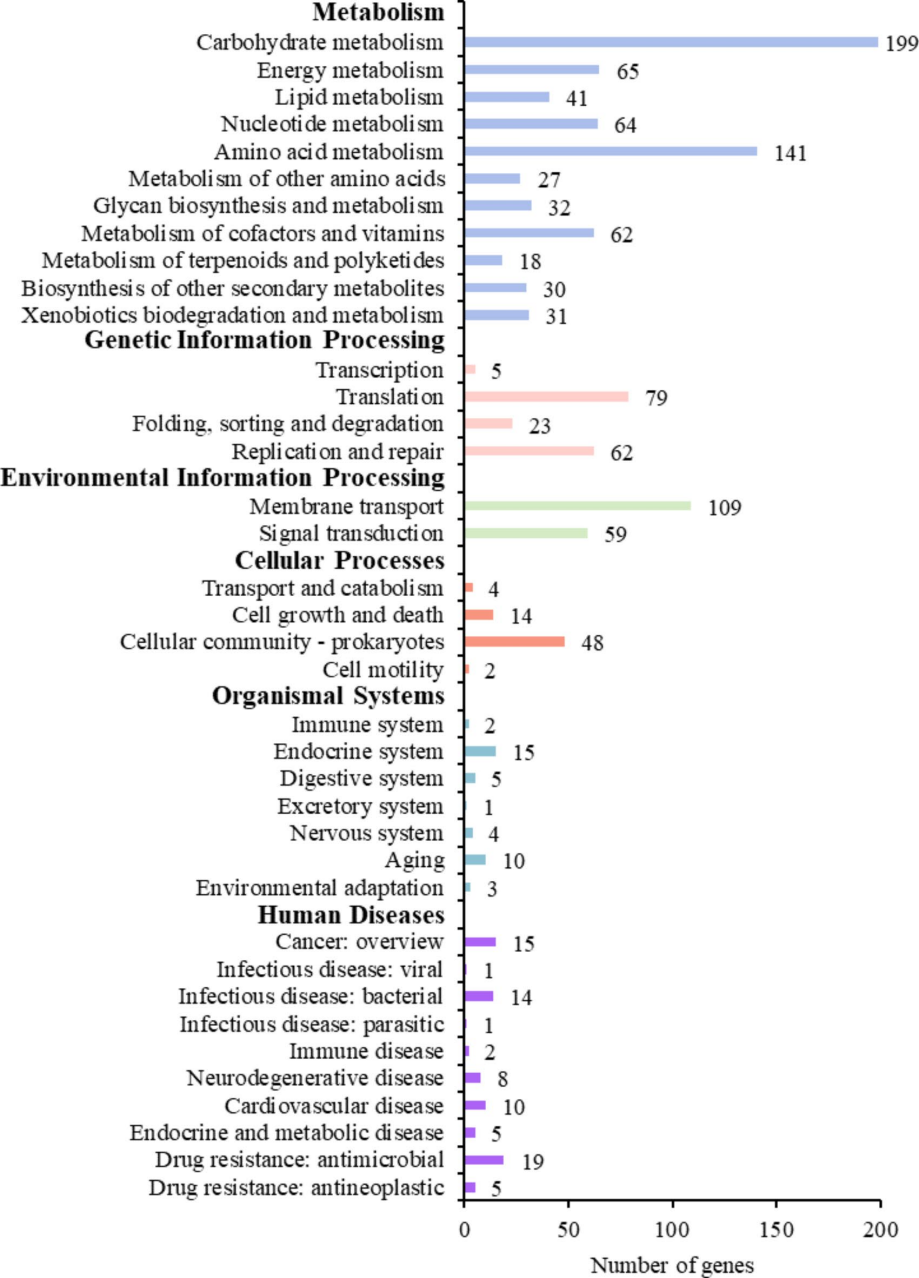
KEGG functional annotation of genes encoded by *Floricoccus penangensis* ML061-4 which is separated in six groups including metabolism (blue), genetic information processing (pink), environmental information processing (green), cellular processes (orange), organismal systems (turquoise) and human diseases (purple).

A total of 1,235 genes (59.9%) harboured by the *F. penangensis* ML061-4 genome were classified into six KEGG functional categories (Fig. 4; Supplementary Table S2). KEGG functional annotation identified genes associated with metabolism (34.4%), genetic information processing (8.2%), environmental information processing (8.2%), cellular processes (3.3%), organismal systems (1.9%) and human diseases (3.9%). Among these, 199 genes (9.7%) were predicted to be associated with carbohydrate metabolism. Biosynthesis of substrates and products is necessary for body structure, microbial survival, replication and evolution^19^. Although gene annotation of *F. penangensis* ML061-4 revealed a number of genes functionally associated with human diseases, this does not mean that the strain is a pathogen.

Metabolic pathways associated with carbohydrate utilization in *F. penangensis* ML061-4 are listed in Supplementary Table S3. The ML061-4 genome is predicted to encode a fructose-bisphosphate aldolase (locus tag number KIW23_01985), a key enzyme of the Embden-Meyerhof (glycolysis) pathway, and an essential enzyme to generate pyruvate from glucose prior to conversion into L-lactic acid via L-lactate dehydrogenase (locus tag number KIW23_05220). No D-lactate dehydrogenase-encoding gene, which would be expected to be responsible for D-lactic acid production, was identified in the ML061-4 genome, in agreement with Rungsirivanich et al.^2^. , who reported that this strain produces a high proportion of L-lactic acid (94.6%).

In vitro carbohydrate fermentation abilities by *F. penangensis* ML061-4 have previously been reported, showing that this strain is able to ferment various monosaccharides (e.g., glucose, fructose and mannose), disaccharides (e.g., lactose, maltose, sucrose and trehalose), oligosaccharide (raffinose), polysaccharide (starch) and a sugar alcohol (mannitol)^1,2^. These results are consistent with KEGG functional prediction of the *F. penangensis* ML061-4 genome. The genome of ML061-4 was also shown to encompass genes required for various phosphoenolpyruvate: sugar phosphotransferase systems (PEP-PTSs), which are known to be involved in the transport and metabolism of carbon sources such as fructose, galactose, glucose, lactose and sucrose^20,21^. The *ptsP* and *ptsH* genes of *F. penangensis* ML061-4 encode the general PTS proteins EI and Hpr, respectively, while genes encoding putative substrate-specific permeases (EII) were also identified, for example *treP* (positions 1,580,662–1,582,716) responsible for the production of EIIBC^trehalose^, and *manA* (positions 1,613,369–1,614,310) encoding a putative EIIABCD^[mannose22^.

**Fig. 5:**
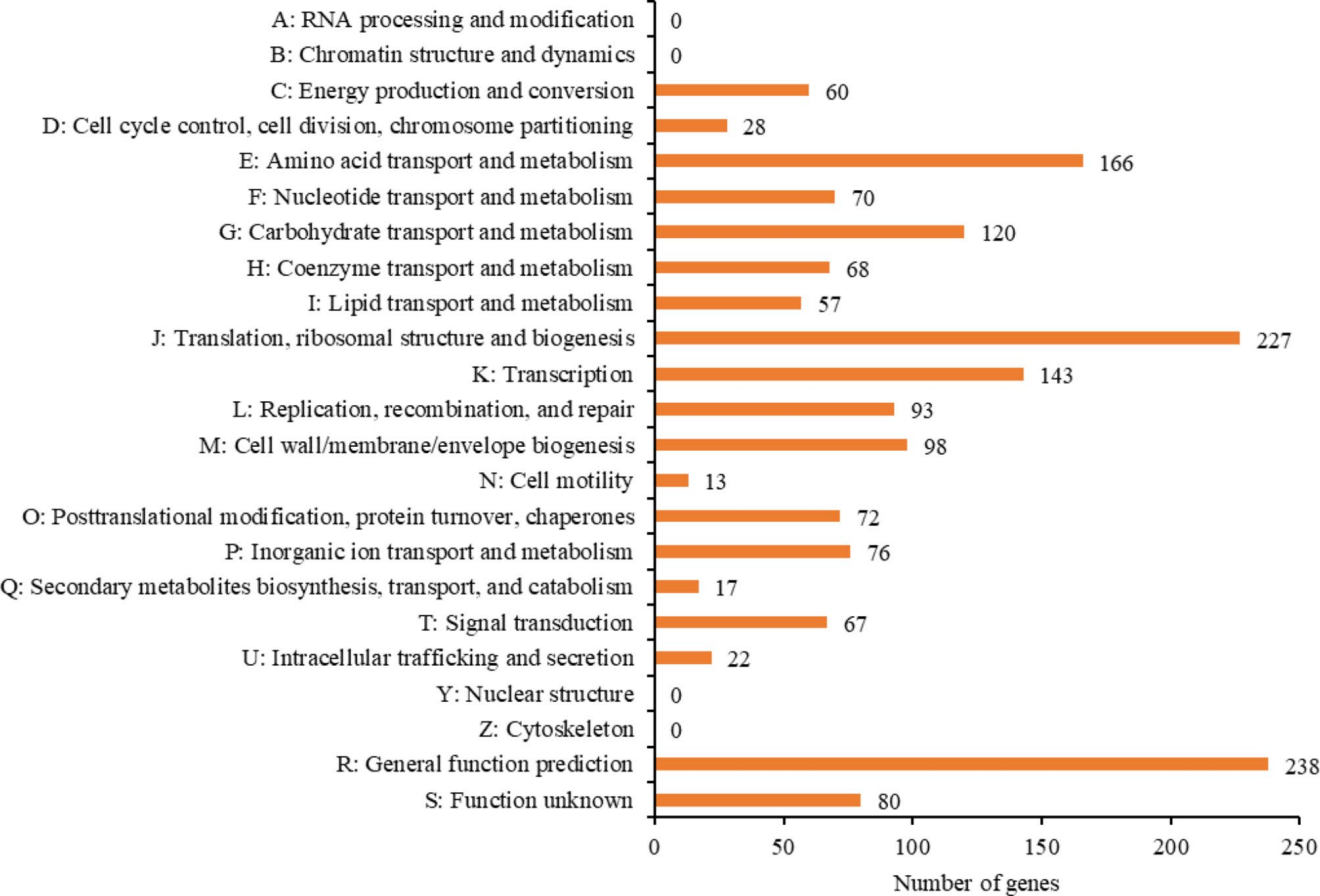
COG functional annotation of genes encoded by *Floricoccus penangensis* ML061-4. Protein-coding sequences are classified according to COG functional categories. Alphabets A-Z exhibits COG functions. A: RNA processing and modification, B: Chromatin structure and dynamics, C: Energy production and conversion, D: Cell cycle control, cell division, chromosome, E: Amino acid transport and metabolism, F: Nucleotide transport and metabolism, I: Lipid transport and metabolism, J: Translation, ribosomal structure and biogenesis, K: Transcription, L: Replication, recombination, and repair, M: Cell wall/membrane/envelope biogenesis, N: Cell motility, O: Posttranslational modification, protein turnover, chaperones, P: Inorganic ion transport and metabolism, Q: Secondary metabolites biosynthesis, transport, and catabolism, R: General function prediction, S: Function Unknown, T: Signal transduction, U: Intracellular trafficking and secretion, Y: Nuclear structure, Z: Cytoskeleton.

**Fig. 6:**
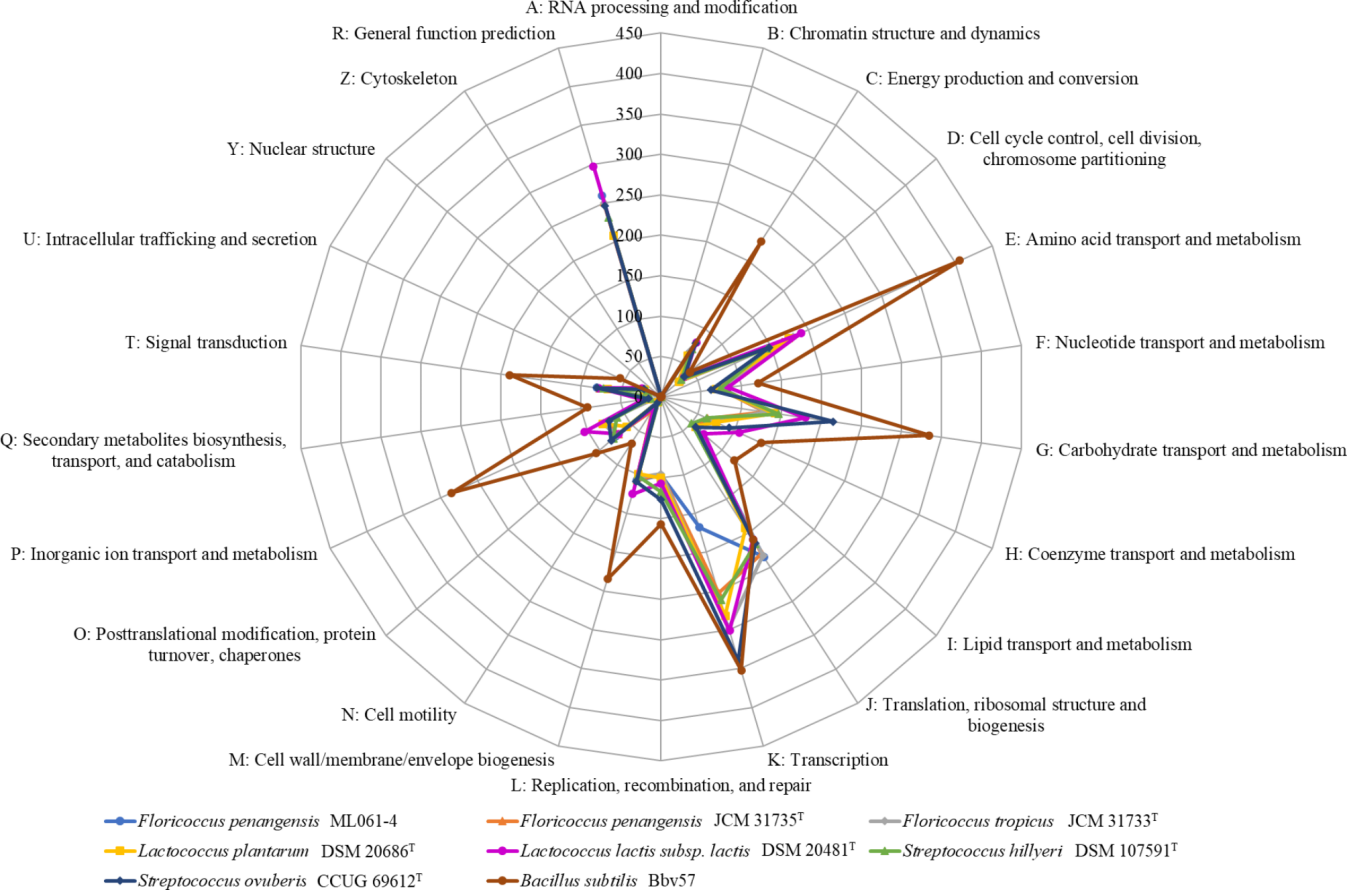
COG functional classification of genes encoded by *Floricoccus penangensis* ML061-4 compared to *F. penangensis* JCM 31735^T^, *F. tropicus* JCM 31733^T^, *L. plantarum* DSM 20686^T^, *L. lactis* subsp. *lactis* DSM 20481^T^, *S. hillyeri* DSM 107591^T^ and *S. ovuberis* CCUG 69612^T^. COG functional classification of genes encoded by *Bacillus subtilis* Bbv57, as previous described by Thiruvengadam et al.^24^. , is used as an outgroup. Between 80 and 170 genes of the genera *Floricoccus*, *Lactococcus* and *Streptococcus* were assigned as function unknown (category S), whereas *Bacillus subtilis* had 1,086 genes assigned to category S.

Furthermore, our functional prediction efforts assigned 1,419 genes (68.8%) to a putative function by the COG database. We found that 16% of the genes identified on the ML061-4 genome was classified into translation, ribosomal structure, and biogenesis (J) category, 11.7% was associated with amino acid transport and metabolism (category E), and 8.5% was involved in carbohydrate transport and metabolism (category G) (Fig. 5; Supplementary Table S4). Comparison of COG functional classification of genes encoded by *F. penangensis* ML061-4, *F. penangensis* JCM 31735^T^, *F. tropicus* JCM 31733^T^, *L. plantarum* DSM 20686^T^, *L. lactis* subsp. *lactis* DSM 20481^T^, *S. hillyeri* DSM 107591^T^ and *S. ovuberis* CCUG 69612^T^ revealed the close phylogenetic relationship between the genera *Floricoccus*, *Lactococcus* and *Streptococcus*. Meanwhile, *Bacillus subtilis* Bbv57, an outgroup, presented higher putative function genes except category J which related to floricoccal, lactococcal and streptococcal members, and disappeared with general function prediction (R) category. Between 80 and 170 genes of the genera *Floricoccus*, *Lactococcus* and *Streptococcus* were assigned as function unknown (category S), a much lower number compared to for example *Bacillus subtilis* strain Bbv57 which had 1,086 genes assigned to this category (Fig. 6; Supplementary Table S5 – S10). This phenomenon is likely due to differences in genome size (flori-, lacto- and streptococcus members possess genomes of approximately 2 Mbp in size (Table 1), whereas bacilli elicit genome sizes that are larger than 4 Mbp)^23,24^.

### Virulence factors, toxin genes and bacterial pathogenicity in *F. penangensis* ML061-4

To identify possible virulence factors and toxins that may be encoded by the ML061-4 genome, a similarity search was performed employing the virulence factors of pathogenic bacteria database (VFDB). Gene clusters with nucleotide sequence similarity values below 80% and E-value above 1e^− 10^ were deemed non-significant^25^. Using these criteria no homologs corresponding to toxin production were found in the genome of ML061-4. The analysis revealed genes associated with virulence factors including *clpC*, *cpsI*, *lap*, *clpP*, *htpB*, *glf*, *tufA*, *groEL*, *hasC* and *clpE* with a nucleotide identity level of 79–90% (Table 2). These genes, many of which represent cellular housekeeping functions, are not necessarily linked directly to virulence such as *clpC* and *htpB* involved in ATP binding, while *cpsI* and *glf* encode UDP-galactopyranose mutase involved in cell wall biosynthesis^26^. Several genes were considered as conserved genes involved in stress responses (*clpC*, *clpP*, *clpE*, *tufA*, *htpB* and *groEL*). Furthermore, PathogenFinder software was applied in order to estimate the bacterial pathogenicity of *F. penangensis* ML061-4. Two genes were predicted to belong to pathogenic protein families including 30S ribosomal protein S19 (*rpsS* gene) and 30S ribosomal protein S21 (*rpsU* gene) with 100.0 and 93.1% identity (Supplementary Table S11). *rpsS* and *rpsU* genes have been described to be associated with rRNA binding, structural constituent of ribosome and tRNA binding (Uniport ID: P0A7U3 and P68679)^27^.

**Table 2:** Prediction of genes related to virulence factor in *F. penangensis* ML061-4 using VFDB database.

VFDB Gene ID	Gene name	Product	Function	Genome location	Nucleotide identity (%)	E-value	NCBI reference sequence number
VFG000079	*clpC*	Endopeptidase Clp ATP-binding chain C	Stress survival	301,586 − 301,743	84	8e-25	NP_463763
VFG002182	*cpsI*	UDP-galactopyranose mutase	Immune modulation	183,518 − 183,732	80	3e-18	WP_002376666
VFG006717	*lap*	*Listeria* adhesion protein Lap	Adherence	1,998,874- 1,998,938	90	3e-12	NP_465159
VFG000077	*clpP*	ATP-dependent Clp protease proteolytic subunit	Stress survival	290,069–290,161	86	3e-12	NP_465991
VFG001855	*htpB*	Hsp60, 60 K heat shock protein HtpB	Adherence	1,720,195- 1,720,355	81	3e-12	WP_197535493
VFG001967	*glf*	UDP-galactopyranose mutase	Immune modulation	183,518 − 183,603	86	2e-10	YP_002344822
VFG046465	*tufA*	Elongation factor Tu	Adherence	640,452–640,588	81	7e-10	WP_003028672
VFG012095	*groEL*	Chaperonin GroEL	Adherence	1,721,173- 1,721,373	79	7e-10	WP_003435012
VFG000964	*hasC*	UTP-glucose-1-phosphate uridylyltransferase HasC	Immune modulation	1,810,158- 1,810,261	82	6e-07	WP_010922799
VFG000080	*clpE*	ATP-dependent protease	Stress survival	1,801,366- 1,801,457	83	6e-07	NP_464522

### Molecular mechanisms associated with adhesion

Four protein-encoding sequences in the ML061-4 genome were predicted to be involved in adhesion based on the VFDB database, including homologs of the *Listeria* adhesion protein (LAP; *lap* gene), 60 K heat shock protein (Hsp60; *htpB* gene), elongation factor Tu (*tufA* gene) and chaperonin (*groEL* gene) with 79–90% nucleotide identity (Table 2). It should be noted that these genes are widely considered to represent housekeeping genes and their physiological role in adhesion has been controversial^28–30^. Previous studies by Burkholder and Bhunia^31^ and Drolia et al.^32^. revealed the roles of LAP and Hsp60 which have been associated with bacterial adhesion ability of *Escherichia coli* and *Listeria monocytogenes* to Caco-2 cells^33^, while chaperonin GroEL and elongation factor Tu were described to participate in mucus adhesion^16,34^. Obtained data corresponding to in vitro experiments by Rungsirivanich et al.^2^. showed that *F. penangensis* ML061-4 possesses adhesion ability to an African green monkey kidney cell line (Vero cell) similar to *Lactobacillus acidophilus* and *Lactiplantibacillus plantarum* strains. The adhesion of microbes to host mucus and epithelial cells is the first step of human colonization and infection^35,36^. Bacterial adhesion ability is considered to be an essential trait to colonize and possible invasion of a host in case of pathogenic bacteria. Conversely, candidate probiotic strains with adhesion features may not only act as competitive exclusion to pathogens, yet may also positively modulate the immune response^37^.

### Bacteriophages, antibiotic and antiviral defenses

To predict prophage elements carried by the *F. penangensis* ML061-4 genome, in silico analysis using PHASTEST software was performed. One region with homology to *Streptococcus* phage PH15 were identified in the genome of *F. penangensis* ML061-4. This 16.1 Kbp DNA region with a 32.85% GC content was predicted to represent an incomplete prophage (Table 3; Supplementary Fig. S1 and S2). Of note, a prophage-encompassing region homologous to the complete *Enterococcus* phage phiFL1A was predicted to be present in the *F. penangensis* JCM 31735^T^ genome, while two prophage regions related to *Lactococcus* phage 50101 and *Bacillus* phage BCJA1c were predicted to be harbored by the genome of *F. tropicus* JCM 31733^T^. Furthermore, various predicted (partial) prophage-encompassing regions were predicted in the *L. lactis* DSM 20481^T^, *S. salivarius* NCTC 8618^T^ and *S. hillyeri* DSM 107591^T^ genomes, while no prophages or remnants thereof appeared to be present in the genomes of *L. plantarum* DSM 20686^T^ and *S. ovuberis* CCUG 69612^T^ (Table 3). None of these predicted prophage regions appeared to be intact; however, the relatedness of these regions to phages of diverse bacterial species provides insights (especially regarding phage origins and evolution) into the co-existence and interactions with other organisms in its environmental niche. Phages can infect bacteria and integrate their genomes into the bacterial genome in order to allow phage multiplication. Such integrated phage genomes or prophages may in turn be activated, resulting in phage particle production and host death^38^. Prophage regions are commonly present in bacterial genomes and have been reported to have many beneficial distinguishing qualities for bacteria such as bacterial adaption in a new environment as well as promoting bacterial resistance^26,39^.

**Table 3:** Gene cluster identification of prophages in *F. penangensis* ML061-4 genome compared with *Floricoccus* spp., *Lactococcus* spp. and *Streptococcus* spp. using PHASTEST.

Strain	Number of phage found	Location	Length (Kbp)	Completeness	GC (%)	Number of phage gene found	Predicted phage	NCBI reference sequence number
*F. penangensis* ML061-4	1	86,797–102,892	16.1	Incomplete	32.85	14	*Streptococcus* phage PH15	NC_010945
*F. penangensis* JCM 31735^T^	1	936,118–1,318,224	48.1	Intact	33.11	24	*Enterococcus* phage phiFL1A	NC_013646
*F. tropicus* JCM 31733^T^	2	703,783–1,097,746	35.7	Intact	33.36	40	*Lactococcus* phage 50101	NC_031040
		1,423,859–1,539,006	44.3	Intact	32.69	40	*Bacillus* phage BCJA1c	NC_006557
*L. plantarum* DSM 20686^T^	Not detected	-	-	-	-	-	-	-
*L. lactis* DSM 20481^T^	2	1,706,646–1,765,449	58.8	Intact	35.40	53	*Lactococcus* phage PLgT-1	NC_031016
		1,951,310–1,976,355	25.0	Incomplete	33.98	14	*Lactococcus* phage bIL312	NC_002671
*L. taiwanensis* NBRC 109049^T^	Not detected	-	-	-	-	-	-	-
*S. salivarius* NCTC 8618^T^	1	666,756–715,649	48.9	Intact	42.02	52	*Streptococcus* phage 5093	NC_012753
*S. hillyeri* DSM 107591^T^	2	1,236,759–1,307,534	38.8	Intact	40.28	47	*Streptococcus* phage 20617	NC_023503
		1,648,481–1,689,670	37.9	Intact	39.78	44	*Streptococcus* phage PH10	NC_012756
*S. ovuberis* CCUG 69612^T^	Not detected	-	-	-	-	-	-	-

To identify genes involved in antibiotic resistance, the complete genome of ML061-4 was determined using CARD database based on RGI criteria with perfect and strict hits. The results showed that the ML061-4 genome did not contain any obvious antibiotic resistance genes. Nevertheless, the KEGG ontology of ML061-4 revealed genes involved in drug resistance pathways including beta-lactam, vancomycin and cationic antimicrobial peptide. A previous study by Rungsirivanich et al.^2^. has shown that *F. penangensis* ML061-4 is susceptible to various tested antibiotics such as ampicillin, cefotaxime, gentamicin, meropenem and tetracycline. The absence of antibiotic resistance genes is suggested to be a desirable property as it eliminates the risk of horizontal transfer to other microorganisms leading to antibiotic drug resistance^40,41^. The genome of *F. penangensis* ML061-4 was shown to lack any CRISPR-Cas systems. CRISPR-Cas systems are found in approximately 40% of assessed bacteria and are known to provide immunity against exogenous nucleic acids, especially bacteriophages^42^. Antiphage systems of strain ML061-4 were predicted by the PADLOC analysis. The results revealed that the genome of *F. penangensis* ML061-4 contains 5 antiphage systems (DMS_other, RM_type_IV, RM_type_IIG, retron_III-A and PD-T4-6) and 2 phage defense candidate systems (PDC-S30 and PDC-M01). Antiphage systems are multigene (located in operons) or single genes. DNA modification systems (DMS) and restriction-modification systems (RM), retrons have been found to play an important role in bacterial antiviral defense^43,44^. The predicted antiviral defence systems of *F. penangensis* ML061-4 are listed in Table 4, Supplementary Fig. S3 and Supplementary Table S12.

**Table 4:** Antiviral defence systems detected in *F. penangensis* ML061-4 genome using PADLOC analysis.

System	Protein name	Genome location	Strand	Relative position
DMS_other	Control_protein	108,671–108,886	Forward	99
DMS_other	DrmMII	108,971–110,209	Forward	100
RM_type_IV	mREase_IV	403,002–405,881	Forward	370
RM_type_IIG	REase_MTase_IIG	713,307–716,267	Forward	646
DMS_other	MTase_II	716,260–717,891	Forward	647
retron_III-A	PRTase_III-A	909,798–911,384	Forward	848
retron_III-A	RT_III-A	911,645–912,646	Forward	850
PDC-S30	PDC-S30	1,106,895–1,107,746	Forward	1042
PD-T4-6	PD-T4-6	1,583,628–1,585,646	Reverse	1507
PDC-M01	PDC-M01B	1,793,808–1,795,058	Reverse	1718
PDC-M01	PDC-M01A	1,795,003–1,797,120	Reverse	1719

### Secondary metabolite biosynthesis and xenobiotics biodegradation

Secondary metabolic potential of strain ML061-4 was examined using antiSMASH, yet did not produce any credible candidates. The antiSMASH software is widely used to predict microbial secondary metabolites for biosynthetic gene clusters particularly antimicrobial, non-ribosomally synthesized peptides (NRPs), polyketides (PKs) and bioactive compounds^45^. Conversely, KEGG ontology of strain ML061-4 revealed 48 genes involved in 20 metabolic pathways for terpenoids and polyketides, and secondary metabolite biosynthesis pathways (e.g., antibiotics, insect hormone and plant secondary metabolites) and 31 genes associated with 11 pathways of xenobiotic biodegradation (e.g., organic compounds and drugs) (Supplementary Table S13). Xenobiotics are chemical substances that are not naturally produced or found in organisms or the environment^46^. In general, xenobiotic refers to environmental pollutants resulting in industry and agriculture such as pesticides, polycyclic aromatic hydrocarbons, pharmaceutical active compounds and phenolic compounds^47^. Xenobiotic biodegradation ability may assist a microorganism to survive in particular polluted environments^48^. KEGG annotation revealed gene clusters involved in xenobiotic biodegradation pathway in ML061-4 genome comprising benzoate, aminobenzoate, chloroalkane, chloroalkene, chlorocyclohexane, chlorobenzene, xylene, styrene, dioxin and naphthalene (Supplementary Table 13). Therefore, secondary metabolites and substances related to xenobiotic biodegradation produced by *F. penangensis* ML061-4 should be further elucidated.

## Materials and methods

### Bacterial strain and growth conditions

*F. penangensis* ML061-4 (GenBank accession no. MH050697) was isolated from the leaf surface of an Assam tea plant (*Camellia sinensis* var. *assamica*) in the Sakat sub-district, Pua district, Nan province, Thailand at an altitude of 1,038 m above sea level (19°15’53.62"N, 101°0’30.22"E)^2^. The *F. penangensis* ML061-4 was cultivated in M17 (Oxoid™, Basingstoke, England) supplemented with 0.5% (w/v) glucose (GM17) broth and incubated at 30 °C for 24 h, as previously reported by Rungsirivanich et al.^23^.

### Genome sequencing and assembly

Whole genome sequencing and assembly of strain Ml061-4 was accomplished as previously described by Rungsirivanich et al.^14^. The genome sequence was deposited in GenBank under accession number CP075561 and Sequence Read Archive (SRA) number SRP399917.

### PCR fingerprinting using the (GTG)_5_ primer

(GTG)_5_-PCR DNA fingerprint analysis of *F. penangensis* ML061-4 was performed according to the protocol of Parlindungan et al.^49^. *Streptococcus thermophilus* Rico66, *S. thermophilus* Brie28, *Lactococcus lactis* Tempeh6L and *L. lactis* RMN5F as previously described by Parlindungan et al.^50^ , *Bacillus velezensis* ML122-2, *B. licheniformis* ML075-1, *B. cereus* TISTR 687 and *B. subtilis* NCTC 10073 as previously described by Rungsirivanich et al.^23^. were used for comparison purposes. PCR amplifications were performed using *Taq* DNA polymerase mastermix (Qiagen, Manchester, UK) with the single oligonucleotide primer (GTG)_5_, 5′-GTGGTGGTGGTGGTG-3′. The PCR reaction mixtures were initially denatured at 95 °C for 7 min, followed by 30 cycles of denaturation at 90 °C for 30 s, annealing at 40 °C for 1 min, extension at 65 °C for 8 min and a final extension step at 65 °C for 16 min using an Applied Biosystems™ 2720 Thermal Cycler (Thermo Fisher, CA, USA). Amplicons were subjected to electrophoresis on a 1% (w/v) agarose gel consisting of ethidium bromide for 1 h at 110 V 100 mA in 1X Tris-acetate-EDTA (TAE) buffer (40 mM Tris-acetate, 1 mM EDTA, pH 8.0). GeneRuler DNA Ladder Mix (Thermo Fisher, CA, USA) was used as a marker. The GTG PCR profiles were visualized under ultraviolet light by gel documentation system, and gel images were captured using GeneSnap software (Syngene, MD, USA).

### Phylogenomic tree analysis

A phylogenomic tree based on whole genome sequences of strain ML061-4 and various members of the genera *Lactococcus* and *Streptococcus* was constructed according to the method of L’Huillier et al.^51^. A total of 16 complete genome sequences were retrieved from the National Centre for Biotechnology Information (NCBI) including *Lactococcus lactis* K_LL005 (GenBank accession no. NZ_CP060580), *Lactococcus lactis* AI06 (GenBank accession no. NZ_CP009472), *Lactococcus lactis* DSM 20481^T^ (GenBank accession no. NC_020450), *Lactococcus cremoris* FM-YL12 (GenBank accession no. CP071728), *Lactococcus cremoris* UC073 (GenBank accession no. CP068698), *Lactococcus protaetiae* KACC 19320^T^ (GenBank accession no. CP041356), *Lactococcus allomyrinae* 1JSPR-7 (GenBank accession no. CP032627), *Lactococcus paracarnosus* TMW 2.1615 (GenBank accession no. CP017195), *Streptococcus parasuis* 1628469 (GenBank accession no. NZ_CP134491), *Streptococcus salivarius* NCTC 8618^T^ (GenBank accession no. NZ_CP009913), *Streptococcus mutans* UA159 (GenBank accession no. AE014133), *Streptococcus sanguinis* CGMH058 (GenBank accession no. CP040798), *Streptococcus toyakuensis* TP1632 (GenBank accession no. NZ_AP024523), *Streptococcus pneumoniae* PJ755/1 (GenBank accession no. CP155535), *Streptococcus pneumoniae* SCAID PHRX1-2021 (GenBank accession no. NZ_CP082820) and *Weissella koreensis* KACC 15510 (GenBank accession no. CP002899). The kSNP 4.0 was applied to perform alignment-free whole genome phylogenetic analysis by identifying single nucleotide polymorphisms (SNPs) across the genome sequences^52^. The obtained SNP-matrix results were imported into MEGA 11 for phylogenetic/phylogenomic tree construction^53^. The phylogenomic tree was analyzed by a Maximum Likelihood method^54^ with bootstrap values of 1000 replicates^55^.

### Genome annotation

Automatic annotation of predicted open reading frames (ORFs) was performed using a combination of Prodigal (version 2.6.3) and BLASTp (version 2.2.26) sequence alignments^56^ to assign annotation, using an E-value cut-off of 0.0001 for hits exhibiting at least 50% similarity across at least 50% of the sequence length) against a non-redundant protein database provided by the NCBI portal. Functional prediction of genes and proteins was integrated using the Clusters of Orthologous Groups (COGs)^57^ and protein family (Pfam)^58^, respectively, as previously described by Martín et al.^59^. Ribosomal RNA (rRNA) and transfer RNA (tRNA) genes were detected using RNAmmer version 1.2^60^ and tRNA-scanSE version 2.0^61^, respectively.

The Kyoto Encyclopedia of Genes and Genomes (KEGG) orthology annotation was undertaken^62^ by the KAAS online server version 2.1^63^ using the bi-directional best hit (BBH) method (https://www.genome.jp/kegg/kaas/). Genome functional characterization was further assigned by BlastKEGG orthology and links annotation (BlastKOALA; https://www.kegg.jp/blastkoala/)^64^. The functional analysis of singleton genes was classified into categories of the COG using the Batch Web CD-Search Tool (https://www.ncbi.nlm.nih.gov/Structure/bwrpsb/bwrpsb.cgi)^65^. The virulence factor search in the ML061-4 genome was conducted using the virulence factors of pathogenic bacteria database (VFDB) (http://www.mgc.ac.cn/VFs/)^66^, and bacterial pathogenicity elements were predicted using PathogenFinder version 1.1 (https://cge.cbs.dtu.dk/services/PathogenFinder/)^67^. Moreover, the presence of lysogenic bacteriophages within the genome was predicted using PHASTEST (https://phastest.ca/)^68^, and putative virulence factors and antibiotic resistance genes were identified by CARD database (https://card.mcmaster.ca/)^69^. Genes involved in secondary metabolite biosynthesis were predicted using antiSMASH (https://antismash-db.secondarymetabolites.org/)^45^. The presence of *c*lustered *r*egularly *i*nterspaced *s*hort *p*alindromic *r*epeats (CRISPR) systems was investigated using CRISPRfinder (https://crispr.i2bc.paris-saclay.fr/Server/)^70^. Antiviral defence systems were identified using PADLOC analysis (https://padloc.otago.ac.nz)^71^.

### Genbank accession numbers and comparative genome analysis

Genome information of *Floricoccus penangensis* JCM 31735^T^ (GenBank accession no. NZ_MKIQ00000000), *Floricoccus tropicus* JCM 31733^T^ (GenBank accession no. NZ_MKIR00000000), *Lactococcus plantarum* DSM 20686^T^ (GenBank accession no. NZ_JXJX00000000), *Lactococcus lactis* DSM 20481^T^ (GenBank accession no. NC_020450), *Lactococcus taiwanensis* NBRC 109049^T^ (GenBank accession no. NZ_BNDT00000000), *Streptococcus salivarius* NCTC 8618^T^ (GenBank accession no. NZ_CP009913), *Streptococcus hillyeri* DSM 107591^T^ (GenBank accession no. NZ_RCVM00000000) and *Streptococcus ovuberis* CCUG 69612^T^ (GenBank accession no. NZ_JAAXPR000000000) was retrieved from the NCBI database (https://www.ncbi.nlm.nih.gov/) for comparative genomic analysis. The genome identity assessments were performed by average nucleotide identity (ANI) determination using EzBioCloud databases (https://www.ezbiocloud.net/tools/ani)^72^.

## Conclusions

This study reports on a functional genomic analysis of a member of the genus *Floricoccus*, and in particular compares its genome to those belonging to closely related members of the genera *Lactococcus* and *Streptococcus*. Comparative genomic analysis showed that the G + C content of the genus *Floricoccus* is explicitly distinguished from *Lactococcus* and *Streptococcus* genera. In silico analysis exhibited that *F. penangensis* ML061-4 encodes enzymes for the metabolism of various carbohydrates. Genome sequence analysis exposed a high level of genetic variation among members of the *Floricoccus*, *Lactococcus* and *Streptococcus* genera. Strain ML061-4 contains genes associated with both probiotic and pathogenic properties, and future research efforts will assist in elucidating the biological functionality, safety and potential biotechnological applications of strains of this species.

## Electronic supplementary material

Below is the link to the electronic supplementary material.

Supplementary Material 1

Supplementary Material 2

## Data Availability

The datasets presented in this study can be found in online repositories. The names of the repository/repositories and accession number(s) can be found at: https://www.ncbi.nlm.nih.gov/genbank/, CP075561, NZ_MKIQ00000000, NZ_MKIR00000000, NZ_JXJX00000000, NC_020450, NZ_BNDT00000000, NZ_CP009913, NZ_RCVM00000000, NZ_JAAXPR000000000, NZ_CP060580, NZ_CP009472, NC_020450, CP071728, CP068698, CP041356, CP032627, CP017195, NZ_CP134491, NZ_CP009913, AE014133, CP040798, NZ_AP024523, CP155535, NZ_CP082820, CP002899.
