# Supplementary material for: Functional genomic insights into Floricoccus penangensis ML061-4 isolated from leaf surface of Assam tea
Source: Sci Rep. 2025 Jan 23;15:2951. doi: 10.1038/s41598-025-86602-x (PMC11758030; doi:10.1038/s41598-025-86602-x)
Supplement: Supplementary file 2 — Supplementary Material 2 [file 41598_2025_86602_MOESM2_ESM.docx]

Supplementary Information

**Functional Genomic Insights into *Floricoccus penangensis* ML061-4 Isolated from Leaf Surface of Assam Tea**

**Patthanasak Rungsirivanich, Elvina Parlindungan, Jennifer Mahony, Witsanu Supandee, Narumol Thongwai & Douwe van Sinderen**


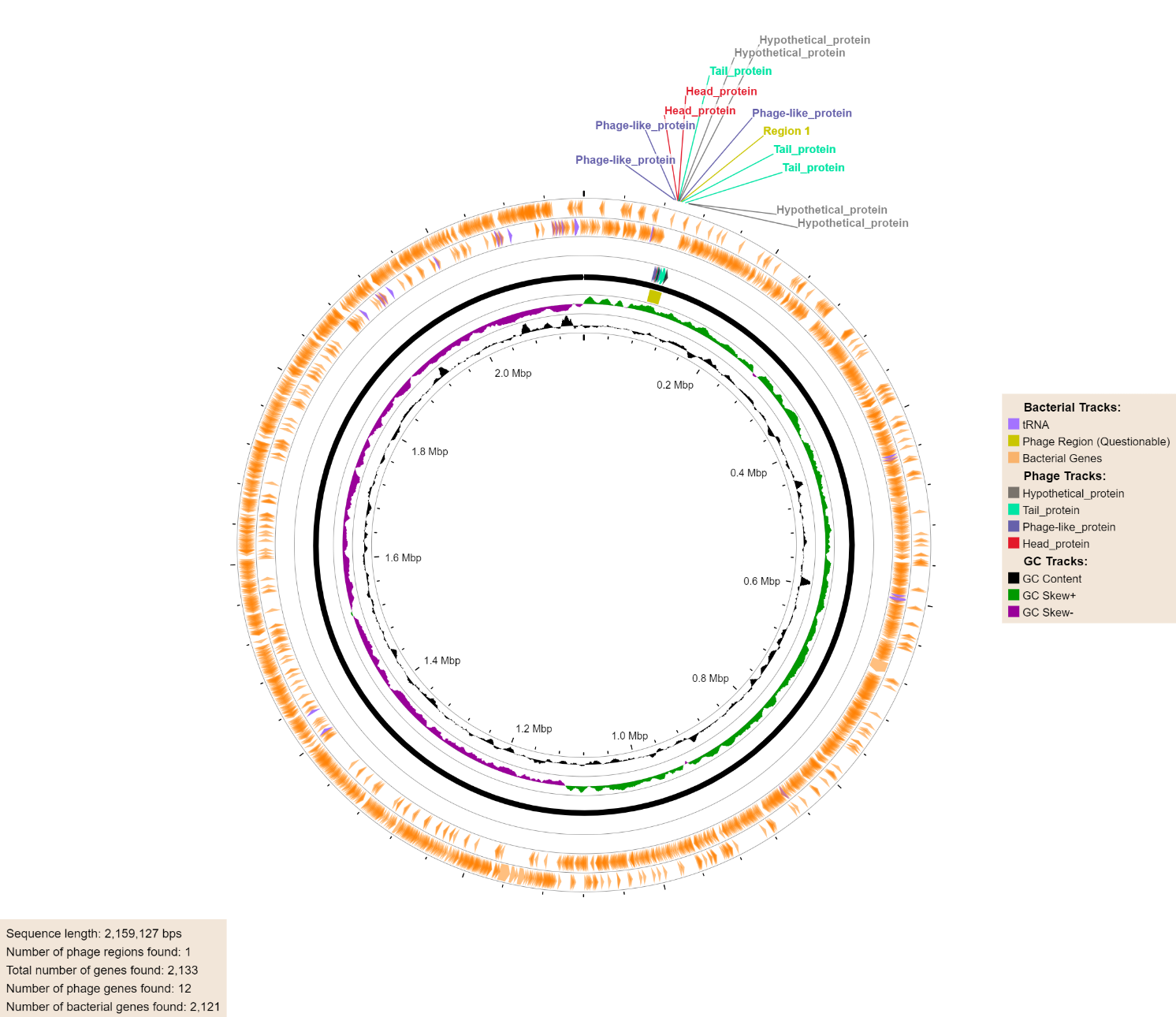


**Supplementary Fig. S1.** The region of proghage elements in the *F. penangensis* ML061-4 genome using PHASTEST.


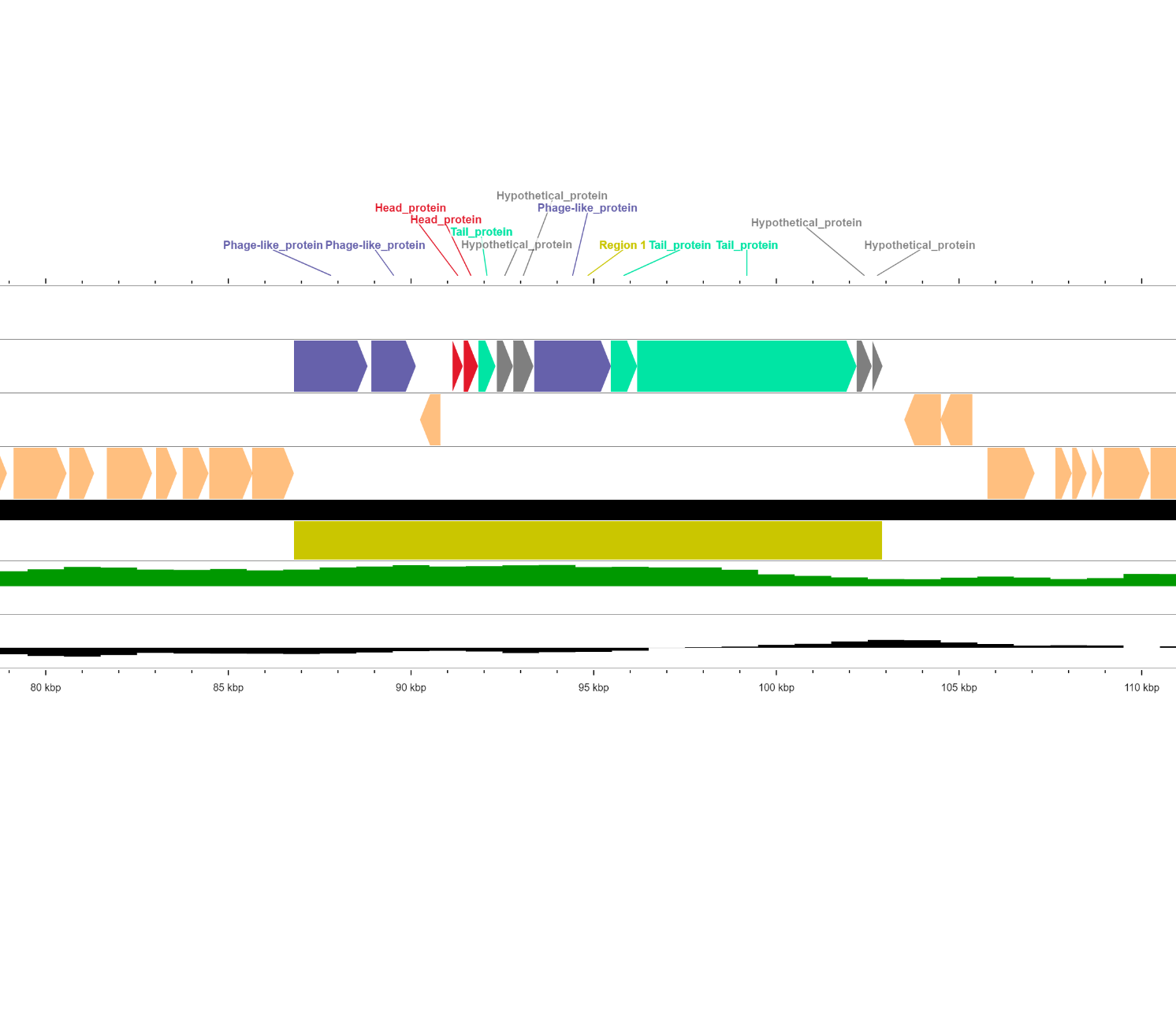


**Supplementary Fig. S2.** Proghage region in the *F. penangensis* ML061-4 genome.


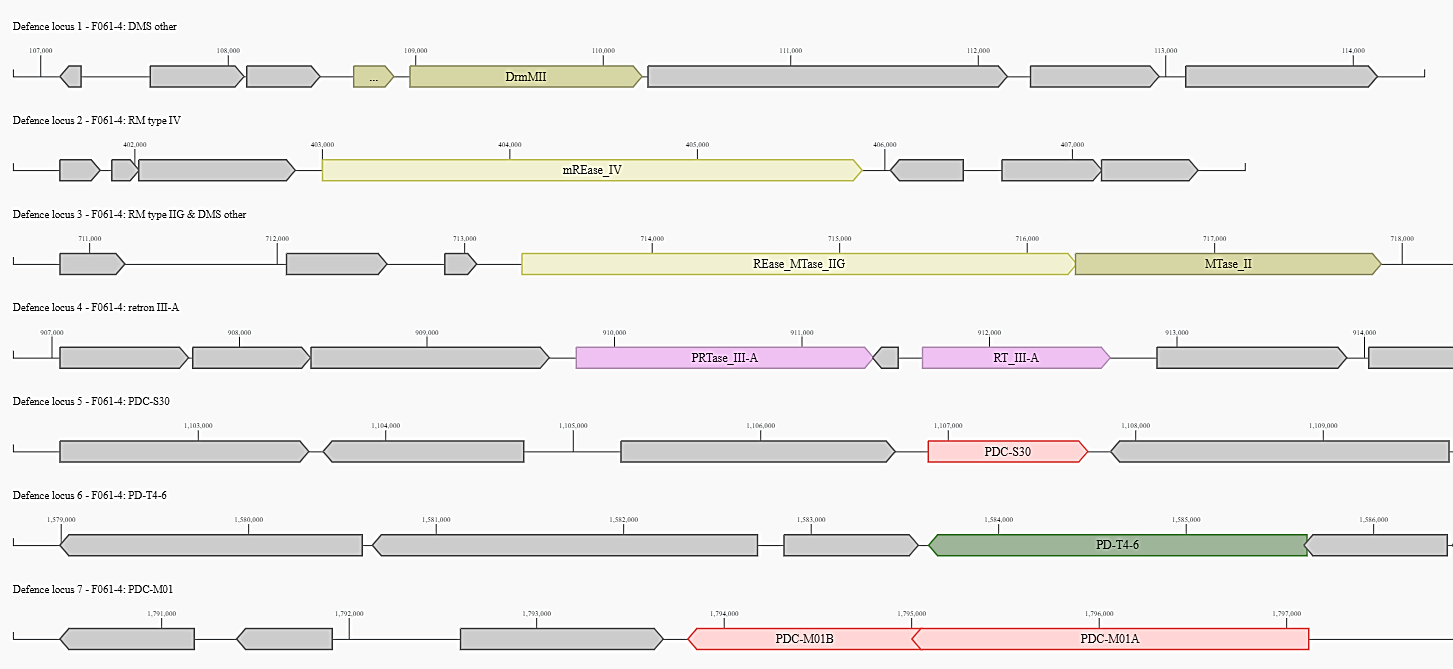


**Supplementary Fig. S3.** Antiviral defence systems detected in the genome of *F. penangensis* ML061-4
